# miR-638 mediated regulation of *BRCA1*affects DNA repair and sensitivity to UV and cisplatin in triple-negative breast cancer

**DOI:** 10.1186/s13058-014-0435-5

**Published:** 2014-09-17

**Authors:** Xiaohui Tan, Jin Peng, Yebo Fu, Shejuan An, Katayoon Rezaei, Sana Tabbara, Christine B Teal, Yan-gao Man, Rachel F Brem, Sidney W Fu

**Affiliations:** 1 Department of Medicine (Division of Genomic Medicine), The George Washington University School of Medicine and Health Sciences, 2300 Eye St. NW, Ross Hall 402C, Washington, 20037 DC USA; 2 Department of Pathology, The George Washington University School of Medicine and Health Sciences, 2150 Pennsylvania Avenue NW, Washington, 20037 DC USA; 3 Department of Surgery, The George Washington University School of Medicine and Health Sciences, 2150 Pennsylvania Avenue NW, Washington, 20037 DC USA; 4 Research Lab and International Collaboration, Bon Secours Cancer Institute, Bon Secours Health System, 5801 Bremo Road, Richmond, 23226 VA USA; 5 Department of Radiology, The George Washington University School of Medicine and Health Sciences, 2300 M St., NW, Room 822, Washington, 20037 DC USA; 6 Department of Microbiology, Immunology and Tropical Medicine, The George Washington University School of Medicine and Health Sciences, 2300 Eye St. NW, Ross Hall 402C, Washington, 20037 DC USA

## Abstract

**Introduction:**

Triple-negative breast cancer (TNBC) represents 15 to 20% of all types of breast cancer; however, it accounts for a large number of metastatic cases and deaths, and there is still no effective treatment. The deregulation of microRNAs (miRNAs) in breast cancer has been widely reported. We previously identified that miR-638 was one of the most deregulated miRNAs in breast cancer progression. Bioinformatics analysis revealed that miR-638 directly targets *BRCA1*. The aim of this study was to investigate the role of miR-638 in breast cancer prognosis and treatment.

**Methods:**

Formalin-fixed, paraffin-embedded (FFPE) breast cancer samples were microdissected into normal epithelial and invasive ductal carcinoma (IDC) cells, and total RNA was isolated. Several breast cancer cell lines were used for the functional analysis. miR-638 target genes were identified by TARGETSCAN-V*ER*T 6.2 and miRanda. The expression of miR-638 and its target genes was analyzed by real-time qRT-PCR and Western blotting. Dual-luciferase reporter assay was employed to confirm the specificity of miR-638 target genes. The biological function of miR-638 was analyzed by MTT chemosensitivity, matrigel invasion and host cell reactivation assays.

**Results:**

The expression of miR-638 was decreased in IDC tissue samples compared to their adjacent normal controls. The decreased miR-638 expression was more prevalent in non-TNBC compared with TNBC cases. miR-638 expression was significantly downregulated in breast cancer cell lines compared to the immortalized MCF-10A epithelial cells. *BRCA1* was predicted as one of the direct targets of miR-638, which was subsequently confirmed by dual-luciferase reporter assay. Forced expression of miR-638 resulted in a significantly reduced proliferation rate as well as decreased invasive ability in TNBC cells. Furthermore, miR-638 overexpression increased sensitivity to DNA-damaging agents, ultraviolet (UV) and cisplatin, but not to 5-fluorouracil (5-FU) and epirubicin exposure in TNBC cells. Host cell reactivation assays showed that miR-638 reduced DNA repair capability in post UV/cisplatin-exposed TNBC cells. The reduced proliferation, invasive ability, and DNA repair capabilities are associated with downregulated *BRCA1* expression.

**Conclusions:**

Our findings suggest that miR-638 plays an important role in TNBC progression via *BRCA1* deregulation. Therefore, miR-638 might serve as a potential prognostic biomarker and therapeutic target for breast cancer.

**Electronic supplementary material:**

The online version of this article (doi:10.1186/s13058-014-0435-5) contains supplementary material, which is available to authorized users.

## Introduction

Breast cancer is the leading cause of cancer-related deaths in women [1]. Clinically, this heterogeneous disease is categorized into four major molecular subtypes: Luminal A, Luminal B, *HER2* type and triple-negative/basal-like. Triple-negative breast cancer (TNBC) constitutes approximately 15 to 20% of all breast cancer cases, with the worst outcome of all subtypes [2]. Systemic treatment for Luminal A and B is based on inhibitors of *ER* signaling, whereas patients with tumors overexpressing *HER2* receptor can be treated with *HER2-*targeting agents. For patients with TNBC, however, there is no targeted therapy available, and chemotherapy has limited duration of effect in later stages of the disease [3].

The precise causes of breast cancer are still unclear. Epigenetic and genetic alterations have long been thought of as two related mechanisms in both the initial development and in breast cancer progression [4]. Breast cancer susceptibility gene 1 (*BRCA1*) is the most well-known gene linked to breast cancer risk [5],[6]. *BRCA1* plays multiple roles in DNA damage response pathways including DNA double-strand break repair, DNA base-excision repair (BER) [7] and nucleotide-excision repair (NER) [8]. Deficiency in *BRCA1* expression tends to exhibit defective DNA repair, which is a critical mechanism of tumorigenesis [9]. *Brca1*-deficient murine mammary epithelial cells are more sensitive to anticancer treatment, such as cisplatin [10].

The crosstalk between the genome and the epigenome offers new possibilities for diagnosis and therapy [11]. Epigenetics has been extended to microRNAs (miRNAs). Mature miRNAs are single-stranded RNA molecules of about 18 to 24 nucleotides, which are endogenously stable and evolutionarily conserved molecules regulating target gene expression [12]. miRNA signatures are associated with clinicobiological features of breast cancer [13],[14]. The advantage of miRNA approaches is based on its ability to concurrently target multiple effectors of pathways. Due to their stability and size, miRNAs can be readily extracted from formalin-fixed, paraffin-embedded (FFPE) samples, or circulating blood as stable markers for cancer detection [15]. miRNA-based anticancer therapies have recently been explored, either alone or in combination with current targeted therapies [16].

Breast cancer is a genetically and phenotypically complex disease [17]. The classic linear multi-step model of breast cancer progression has been observed based on histomorphological and epidemiological data. The earliest neoplastic stage of progression is atypical ductal hyperplasia (ADH), in which molecular alterations occur in breast epithelium of a normal terminal duct lobular unit. Subsequent molecular alterations occur in ADH, resulting in ductal carcinoma *in situ* (DCIS), another early neoplastic stage, upon which additional events occur, resulting in invasive ductal carcinoma (IDC) [18]. In our previous work, we identified deregulated miRNAs in the progression of breast cancer development using FFPE samples from breast cancer tissue. We found that miR-21, miR-200b/c, miR-141, and miR-183 were consistently upregulated in ADH, DCIS and IDC compared to normal, while miR-638 was uniquely downregulated in ADH and DCIS [19]. Differentially expressed miR-638 has been detected in the majority of tumors [20]-[25]. More interestingly, upregulation of miR-638 could be a biomarker in response to DNA damage [26]. In the present study, we aim to understand the molecular mechanisms of miR-638 deregulation in breast cancer by investigating its effects on proliferation, invasion, DNA repair and sensitivity to anticancer drugs/UV light in breast cancer, with a particular focus on TNBC.

## Materials and methods

### FFPE breast cancer samples and microdissection

The tissue blocks were retrieved from the tissue repository of the Armed Forces Institute of Pathology (AFIP) with its IRB (Institutional Review Board) approval. This study was approved by the IRB of the George Washington University. All specimens are anonymized and not coded; therefore they cannot be linked back to the individual subject identities in any way. No consent was needed for this study. The FFPE blocks were subject to microdissection into IDC and normal components as described previously [19].

### Breast cancer cell lines and cell culture

The human breast cancer cell lines, MDA-MB-231, Hs578T, MCF-7 and T47D were purchased from the American Type Culture Collection (ATCC), and cultured in Dulbecco’s modified Eagle’s medium (DMEM) (Lonza Group Ltd, Basel, Switzerland) supplemented with 10% fetal bovine serum (FBS) and 1% penicillin and streptomycin antibiotics. Immortalized MCF-10A cells were cultured in mammary epithelial cell growth medium (MEGM) (CC-3150, Lonza) containing 100 ng/ml of cholera toxin to make a complete growth culture medium. All cell lines were grown in a 37°C humidified incubator with 5% CO_2_.

### Total RNA extraction

Total RNA was isolated from the breast cancer cells, including the transfected lines using the Trizol reagent (Life Technologies, Carlsbad, CA, USA) following the manufacturer’s instructions. The Recover All Total Nucleic Acid Isolation Kit (AM1975, Ambion Diagnostics, Austin, TX, USA) was used to isolate total RNA from the FFPE samples as described earlier [19]. Briefly, 1 ml of xylene was added to four 20 μm FFPE sections to remove paraffin. The tissue was digested with proteinase K at 55°C overnight and then treated with DNase I. After washing, total RNA, including the small miRNA fraction, was reconstituted in distilled water. Quantity and quality of the total RNA samples were assayed by the NanoDrop1000 Spectrophotometer (Thermo Fisher Scientific, Waltham, MA, USA).

### Quantitative real-time reverse transcription-PCR (qRT-PCR) assay

The Taqman MiRNA Reverse Transcript Kit (Applied Biosystems, Foster City, CA, USA), which features a stem-loop RT primer specifically hybridizing with a miRNA was used. The reverse transcription was performed using the MultiScribe Reverse Transcriptase. Specifically, 10 ng of the total RNA was used to start the RT step following the manufacturer’s protocol. The RT reactions were carried out at 16°C for 30 minutes, 42°C for 30 minutes, 85°C for 5 minutes and then held at 4°C. To verify miRNA expression, a final volume of 20 μl for each PCR reaction mixture consisting of 10 μl TaqMan Universal Master Mix II with no UNG (Applied Biosystems), 1 μl of 20 x Taqman miR-638 PCR primer (Ambion), 2 μl of 1:1 diluted RT products and 7 μl nuclease-free water. qPCR was performed using the ABI 7300 Real-Time PCR System (Applied Biosystems). The conditions for qPCR were 95°C for 10 minutes, followed by 40°Cycles of 95°C for 15’seconds and 60°C for 60’seconds. The mean quantity values of the miRNA expression were normalized by U6 snRNA. Primer sequences are available upon request.

### miRNA target analysis

The potential target genes of miR-638 were analyzed using the TARGETSCAN-V*ER*T 6.2 [27] and miRanda, which help identify targets based on comparative sequence analysis, seed match complementation and Z-score for assigned untranslated regions (UTRs) and coding sequence (CDS) region. A group of selected target genes were further analyzed.

### Dual luciferase reporter assay

The 3′-untranslated region (3′-UTR) of the *BRCA1*-wild-type (W) or -mutant (M) was cloned to the firefly luciferase-expressing vector, pEZX-MT05 (Genecopoeia, Rockville, MD, USA). The *BRCA1*-W and -M CDS of miR-638 binding site was constructed in pGL3 plasmid (a gift from Dr. Wen Chen from Sun Yat-sen University, China). For the luciferase assay, cells (7 × 10^5^ cells per well in a 24-well plate) were co-transfected with the 3′UTR or CDS of *BRCA1* reporter vector and miR-638 mimics or the control vector pEZX-MT05 negative-control (mock) using FuGENE Transfection Reagent (Promega, Madison, WI, USA). Luciferase activities were determined with the Dual-Luciferase Reporter System (Genecopoeia). Each sample was measured in triplicate using the Glomax Luminometer (Promega).

### Protein extraction and Western blot analysis

Proteins were extracted from cell lines using RIPA Buffer (Thermo Fisher Scientific) according to the manufacturer's protocol. Proteins were separated by SDS-PAGE using a 4 to 15% Mini-*PR*OTEAN TGX™ Precast Gel (Bio-Rad Laboratories, Hercules, CA, USA) and transferred overnight at 30 V in a 4°C cold room. The membrane was blocked prior to the addition of the primary antibody with 5% milk in Tris-buffered saline (TBS) with 0.05% Tween 20. The membrane was incubated overnight with either *BRCA1* rabbit polyclonal antibody (9010S, Cell Signaling Technology, Danvers, MA, USA) at a dilution of 1:1000 in TBS buffer with 0.05% Tween and 5% milk, or *GAPDH* (MA5-15738) mouse monoclonal antibody (Sigma-Aldrich, St Louis, MO, USA) at a dilution of 1:2,000 in TBS buffer with 0.05% Tween. The membrane was washed three times with TBS/0.05% Tween and incubated with anti-rabbit immunoglobulin G (IgG) conjugated to horseradish peroxidase (7074S, Cell Signaling) for *BRCA1*, anti-mouse IgG (7076S, Cell Signaling) for *GAPDH* at a 1:2,000 dilution in TBS/0.05% Tween and 5% milk. The Super Signal WestFemo Maximum Sensitivity Substrate (Thermo Fisher Scientific) was used according to the manufacturer's protocol to visualize protein expression and the band intensities were quantified by the ImageJ software.

### Transfection of miR-638 mimic and miR-638 inhibitor in human breast cancer cell lines

Using transient transfection, 2.4 × 10^5^ cells of each cell line were seeded in a 6-well plate, cultured in DMEM medium supplemented with 10% FBS in a 37°C humidified incubator with 5% CO_2_. After overnight incubation, cells reached 30% to 50% confluence and were transiently transfected with miR-638 mimic, miR-638 inhibitor or mock (Ambion) by Lipofectamine RNAiMAX reagent (Life Technologies) using the Opti-MEM medium (Life Technologies). Cells were subjected to further analysis after 24 h transfection.

### Matrigel invasion assay

Matrigel invasion assays were performed using the BD BioCoat™ Matrigel™ Invasion Chamber (BD Biosciences, Franklin Lakes, NJ, USA) as previously described [28]. BD BioCoat Matrigel Invasion Chambers provide cells with the conditions that allow assessment of their invasive property *in vitro*. It consists of a BD Falcon™ TC Companion Plate with Falcon Cell Culture Inserts containing an 8 micron pore size PET membrane with a thin layer of matrigel basement membrane matrix. Briefly, prior to the start of each experiment, 500 μl of warm (37°C) serum-free DMEM medium was added to the upper and lower chambers and allowed to rehydrate for 2 h in a 37°C cell culture incubator. After 2 h rehydration, the medium was removed from the upper and lower chambers, 750 μl of DMEM with 10% FBS and 0.1% bovine serum albumin (BSA) was added to the pre-wetted lower chambers. Then 2.5 × 10^4^ cells for MDA-MB-231, 3 × 10^4^ for Hs578T, 5 × 10^4^ for MCF-7, 8 × 10^4^ for T47D, transfected by either miR-638 or mock for 24 h were seeded onto the top chamber of pre-wetted inserts, cultured in 500 μl serum-free DMEM with 0.1% BSA in the top chamber. Cells were incubated in a matrigel chamber in a 37°C humidified incubator with 5% CO_2_ for 24 h for MDA-MB-231 and 48 h for Hs578T, MCF-7 and T47D. Next, the noninvasive cells were removed from the upper surface of the membrane by scrubbing with a cotton swab and the invasive cells present on the bottom of the membrane were fixed, stained with the Diff-Quick staining solution and counted (five microscope fields under the 10X lens). Experiments were done in duplicate for each cell line twice. Cell counts were performed on five non-overlapping random fields for each chamber, and four chambers were counted for each experimental point. The percentage of invasive cells was normalized to the corresponding control.

### UVC/chemosensitivity and MTT assays

The miR-638 mimic or mock transfected cells were washed with phosphate-buffered saline (PBS) 100 μl of MTT working solution (5 mg/ml stock MTT diluted in the Opti-MEM media to 0.5 mg/ml working solution) was added to each well and incubated at 37°C with 5% CO_2_ for 3 h. The MTT solution was carefully removed and 100 μl DMSO was added to each well and incubated in a 37°C humidified incubator with 5% CO_2_ for 30 min. Color development was measured using a spectrophotometer at 570 nm on a plate reader (Bio-Tek Instruments, Winooski, VT, USA) and quantified following the manufacturer’s protocol (Promega). For the MTT chemosensitivity assay, cells were treated with UVC (10 J/m^2^) and various concentrations of cisplatin (0.5 to 8 μg/mL), 5-fluorouracil (5-FU, 5 to 400 μg/mL), or epirubicin (0.025 to 1.6 μg/mL). After 48 h, MTT solution was added and absorbance was measured.

### Plasmid treatment with UV light and anticancer drugs and host cell reactivation assay

pCMVLuc reporter gene plasmid (a kind gift from Dr. Kenneth H. Kraemer, National Cancer Institute, NIH) was dissolved in 10 mm Tris-HCl, 1 mm EDTA, pH 8 (TE buffer) to a final concentration of 100 μg /ml and poured in a petri dish to form a one-dimensional 2 mm thick layer. The petri dish was placed on ice and irradiated by 1,000 J/m^2^ of UV light. For the drug treatment, 1 μl aliquots of a stock solution of 1 μg*/*μl cisplatin, 10 μg*/*μl 5-FU and 0.01 μg*/*μl epirubicin (Sigma-Aldrich) in TE were added to 10 μg plasmid DNA dissolved in 200 μl TE buffer and the samples were incubated at 37°C for the 6 hours. At the end of the incubation period, 1 M NaCl was added to a final concentration of 0.2 M NaCl. The plasmid DNA was precipitated with 2 volumes of ethanol, washed with 70% ethanol before dissolving in TE buffer.

DNA repair capability of cells was assessed using the host cell reactivation (HCR) assay with the pCMVLuc reporter gene plasmid treated by UV or anticancer drugs [29]. Briefly, 4 μl (200 ng) of CsCl-purified pCMVLuc damaged or non-damaged were co-transfected with 50 nM miR-638 mimic into cells using Lipofectamine 2000 (Invitrogen). To estimate the DNA repair capacity after miR-638 knockdown, we co-transfected pCMVLuc with 50 mol of miR-638 inhibitor. Relative luciferase activities are presented as a percentage of activities obtained with treated versus untreated control plasmids.

### Statistical analysis

Data was expressed as mean ± standard error (SE). Permutation test was performed for MTT assay between control and miR-638 mimic transfected groups. The Student’s *t* test (two-tailed) was applied to the matrigel assays between control and miR-638 transfected groups. *P* value less than 0.05 or 0.01 was considered statistically significant and presented with one and two asterisks respectively.

## Results

### Decreased expression of miR-638 in breast cancer

**Figure 1:**
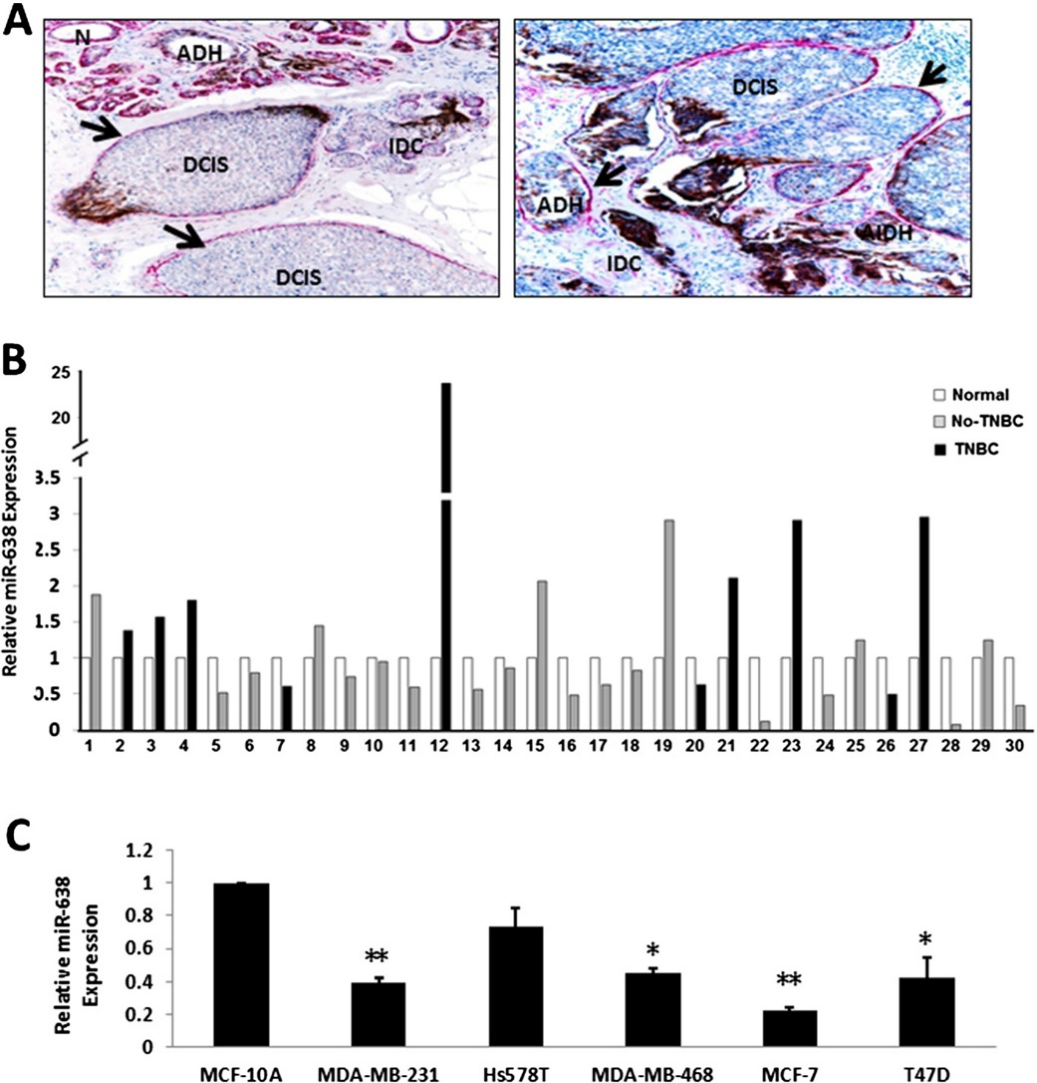
**Expression of miR-638 in breast cancer tissue samples and cell lines. (A)** Representative images before microdissection. Breast cancer tissue sections were double immunostained for smooth muscle actin (red) to elucidate the epithelial capsule (arrows). Different components, such as normal (N), ADH, DCIS and IDC are as indicated. **(B)** Expression of miR-638 in TNBC and non-TNBC cases. Expression of miR-638 was significantly downregulated in 3 of 10 TNBCs and 15 of 20 non-TNBCs when compared to normal. **(C)** Expression of miR-638 in breast cancer cell lines was downregulated compared to the immortalized MCF-10A cells. Results are displayed as mean data ± SE. (*P <0.05 and **P <0.01). ADH, atypical ductal hyperplasia; DCIS, ductal carcinoma *in situ*; IDC, invasive ductal carcinoma; TNBC, triple-negative breast cancer.

In our previous work, we found that miR-638 expression was decreased in both ADH and IDC stages in breast cancer [19]. To further evaluate the relationship between miR-638 expression and progression of breast cancer, we examined miR-638 expression in 30 breast cancer samples after microdissecting into normal and IDC components (Figure 1A) by qRT-PCR. Downregulated miR-638 expression was detected in 18 of 30 (60%) cases, including 15 of 20 (75%) non-TNBC and 3 of 10 (30%) TNBC cases compared with their adjacent normal samples (Figure 1B). Thus, deregulation of miR-638 is more prevalent in non-TNBC compared to TNBC cases.

Next, we analyzed the expression of miR-638 in the following cell lines, including TNBC lines, MDA-MB-231, Hs578T and MDA-MB-468 and non-TNBC lines, MCF-7 and T47D in comparison to the normal immortalized MCF-10A cells. We found that miR-638 expression in all breast cancer cell lines was relatively low compared to that in MCF-10A cells (Figure 1C). This data indicates that miR-638 might be a tumor suppressor in breast cancer.

### miR-638 target gene identification

TargetScan and miRanda were used to identify target genes for miR-638 (miRbase.org website). We obtained a list of target genes with the information pertaining to the binding sites for miR-638, including *BRCA1* (Table 1). To identify the common target genes for miR-638, we narrowed down the functional pathway enrichment analysis using two different algorithms. Since *BRCA1* is a multifunctional tumor suppressor protein and plays multiple roles in DNA damage response pathways, we focused on *BRCA1* as a target gene for this study.

**Table 1:** **A representative list of target genes for miR-638**

Target gene	Representative transcript	Gene name	Conserved sites				Total context + score
			**Total**	**8-mer**	**7mer-m8**	**7mer-1A**	
STARD10	NM_006645	StAR-related lipid transfer (START) domain containing 10	1	1	0	0	−0.53
NPAS4	NM_178864	neuronal PAS domain protein 4	1	1	0	0	−0.44
MKLN1	NM_001145354	muskelin 1, intracellular mediator containing kelch motifs	1	0	0	1	−0.27
CCDC92	NM_025140	coiled-coil domain containing 92	1	0	0	1	−0.26
SHPK	NM_013276	sedoheptulokinase	1	0	0	1	−0.26
RIMKLB	NM_020734	ribosomal modification protein rimK-like family member B	1	0	1	0	−0.24
PGK1	NM_000291	phosphoglycerate kinase 1	1	0	0	1	−0.22
HP1BP3	NM_016287	heterochromatin protein 1, binding protein 3	1	0	0	1	−0.21
LMO4	NM_006769	LIM domain only 4	1	0	0	1	−0.21
PAK2	NM_002577	p21 protein (Cdc42/Rac)-activated kinase 2	1	0	0	1	−0.2
MARCKS	NM_002356	myristoylated alanine-rich protein kinase C substrate	1	0	0	1	−0.19
FRMPD4	NM_014728	FERM and PDZ domain containing 4	1	0	0	1	−0.18
EMILIN3	NM_052846	elastin microfibril interfacer 3	1	0	0	1	−0.18
MYCL1	NM_001033081	v-myc myelocytomatosis viral oncogene homolog 1, lung carcinoma derived (avian)	1	0	0	1	−s0.18
LOC100507050	NM_001195528	hypothetical LOC100507050	1	0	0	1	−0.18
ISOC2	NM_001136201	isochorismatase domain containing 2	1	0	0	1	−0.18
EDN3	NM_207032	endothelin 3	1	0	0	1	−0.18
BRCA1	NM_007294	breast cancer 1, early onset	1	0	0	1	−0.16
TSPAN1	NM_005727	tetraspanin 1	1	0	0	1	−0.16
BUB3	NM_004725	budding uninhibited by benzimidazoles 3 homolog (yeast)	1	0	0	1	−0.15
SP8	NM_182700	Sp8 transcription factor	1	0	0	1	−0.15
ZNF24	NM_006965	zinc finger protein 24	1	0	0	1	−0.14
KDSR	NM_002035	3-ketodihydrosphingosine reductase	1	0	0	1	−0.14
KPNA6	NM_012316	karyopherin alpha 6 (importin alpha 7)	1	0	0	1	−0.13
SP7	NM_001173467	Sp7 transcription factor	1	0	0	1	−0.12
NOVA1	NM_002515	neuro-oncological ventral antigen 1	1	0	0	1	−0.11
SENP1	NM_014554	SUMO1/sentrin specific peptidase 1	1	0	0	1	−0.06
SRSF1	NM_001078166	serine/arginine-rich splicing factor 1	1	0	0	1	−0.05
EPHA7	NM_004440	EPH receptor A7	1	0	0	1	−0.04
TTC28	NM_001145418	tetratricopeptide repeat domain 28	1	0	0	1	−0.04

### miR-638 exerted diverse effects on BRCA1 expression depending upon the subtypes of breast cancer cell lines

**Figure 2:**
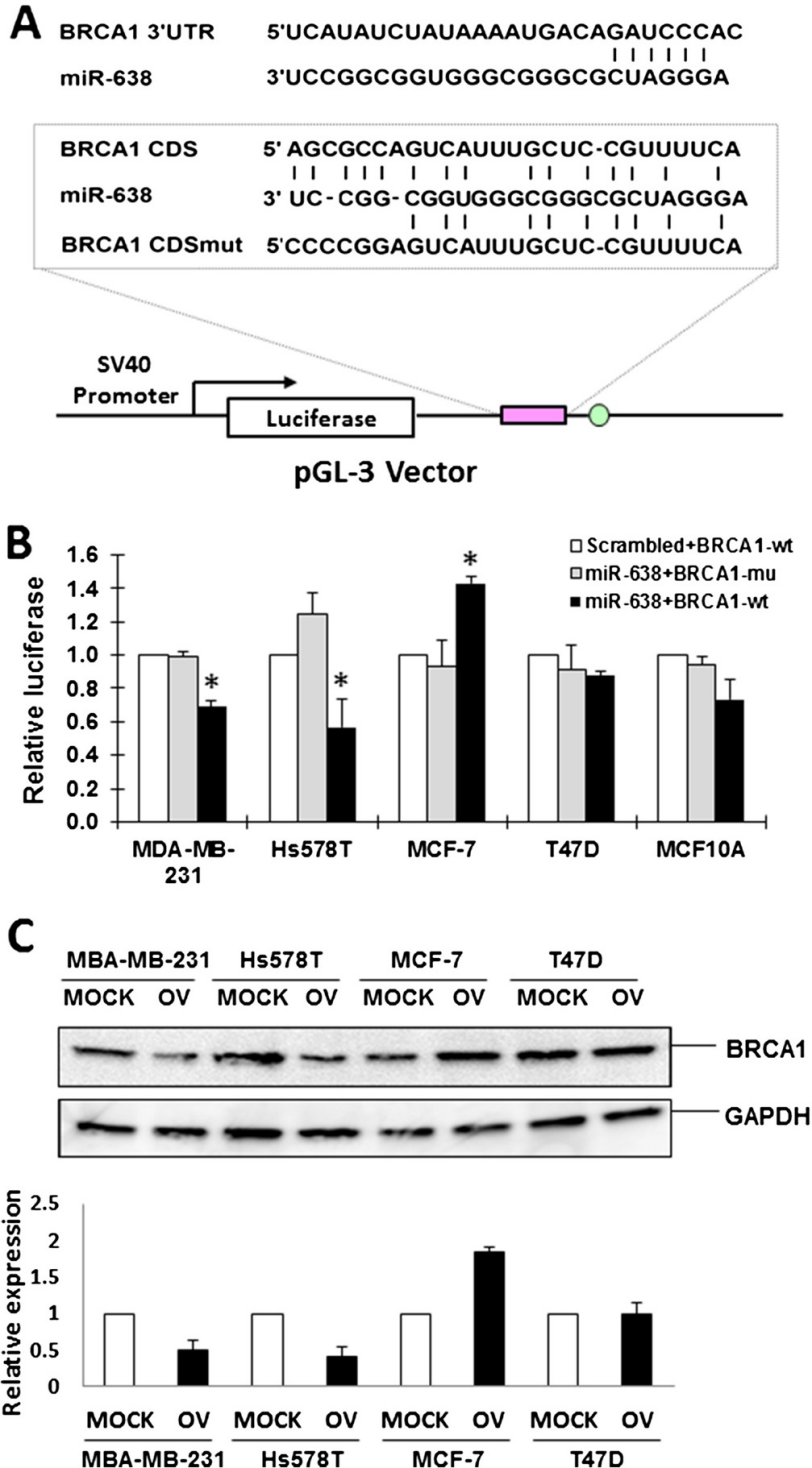
**miR-638-mediated regulation of** ***BRCA1*** **in breast cancer cell lines. (A)** The top part shows the predicted location of the miR-638 binding site of the 3’-UTR of *BRCA1*. The bottom part indicates the binding site in the CDS of *BRCA1* (*BRCA1* CDS) as well as the mutant *BRCA1* sequence (*BRCA1* CDS mut) corresponding to miR-638 sequence, along with the pGL-3 vector. **(B)** Relative luciferase activity was measured in breast cell lines co-transfected with either 200 ng of miR-638 mimic (dark bars) or a scrambled control (white bar), 100 ng of either pGL3-*BRCA1*-CDS (*BRCA1*-wt) or pGL3-*BRCA1*-CDS mut (*BRCA1*-mu) by Lipofectamine 2000 (Life Technologies) for 48 h. The data was reported as mean ± SE for three independent experiments. The luciferase activity was significantly decreased in MDA-MB-231 and Hs578T cells when co-transfected with miR-638 mimic and *BRCA1*-wt (^*^P <0.05), while either increased in MCF-7 or not changed in T47D and MCF-10A cells. **(C)** The expression of *BRCA1* protein in breast cancer cells when transfected with miR-638 mimic (OV) compared to the mock (Mock). *BRCA1* expression was significantly downregulated by miR-638 in TNBC cells, MDA-MB-231 and Hs578T compared to non-TNBC cells, MCF-7 and T47D. The experiments were repeated three times. Band intensities were quantified using the ImageJ software, and the relative expression level was shown in a bar graph. *BRCA1*, breast cancer susceptibility gene 1; CDS, coding sequence; TNBC, triple-negative breast cancer; UTR, untranslated region.

To validate the computational predictions and the biological effect of miR-638 targeting *BRCA1*, we carried out *in vitro* luciferase reporter assays. miR-638 has been reported to inhibit *BRCA1* expression by targeting *BRCA1* in CDS but not in the 3’ UTR [30]. We performed luciferase reporter assay with the pGL3 plasmid containing miR-638-binding site in *BRCA1* CDS region (Figure 2A). We found that the luciferase activities vary in different breast cancer cell lines after successful transfection of miR-638 mimics (Figure 2B). Luciferase activities were significantly decreased in miR-638-transfected TNBC cell lines MDA-MB-231 and Hs578T, but not in estrogen receptor (ER)-positive cell lines T47D and immortalized MCF-10A cells. Inversely, the luciferase activities were significantly increased in miR-638-transfected MCF-7 cells. There was no significant difference in luciferase activities between controls and mutant miR-638 transfectants (Figure 2B). Inconsistent with the luciferase assay results, significant downregulation of *BRCA1* was observed in TNBC cell lines MDA-MB-231 and Hs578T, but upregulation of *BRCA1* was shown in ER-positive cell lines, MCF-7 and T47D (Figure 2C). These results demonstrate that miR-638 may specifically regulate *BRCA1* in TNBC, not in *ER* + cells, which suggests that the function of miR-638 might be blocked or antagonized by hormonal receptor expression.

### Overexpression of miR-638 inhibited proliferation in TNBC cell lines

**Figure 3:**
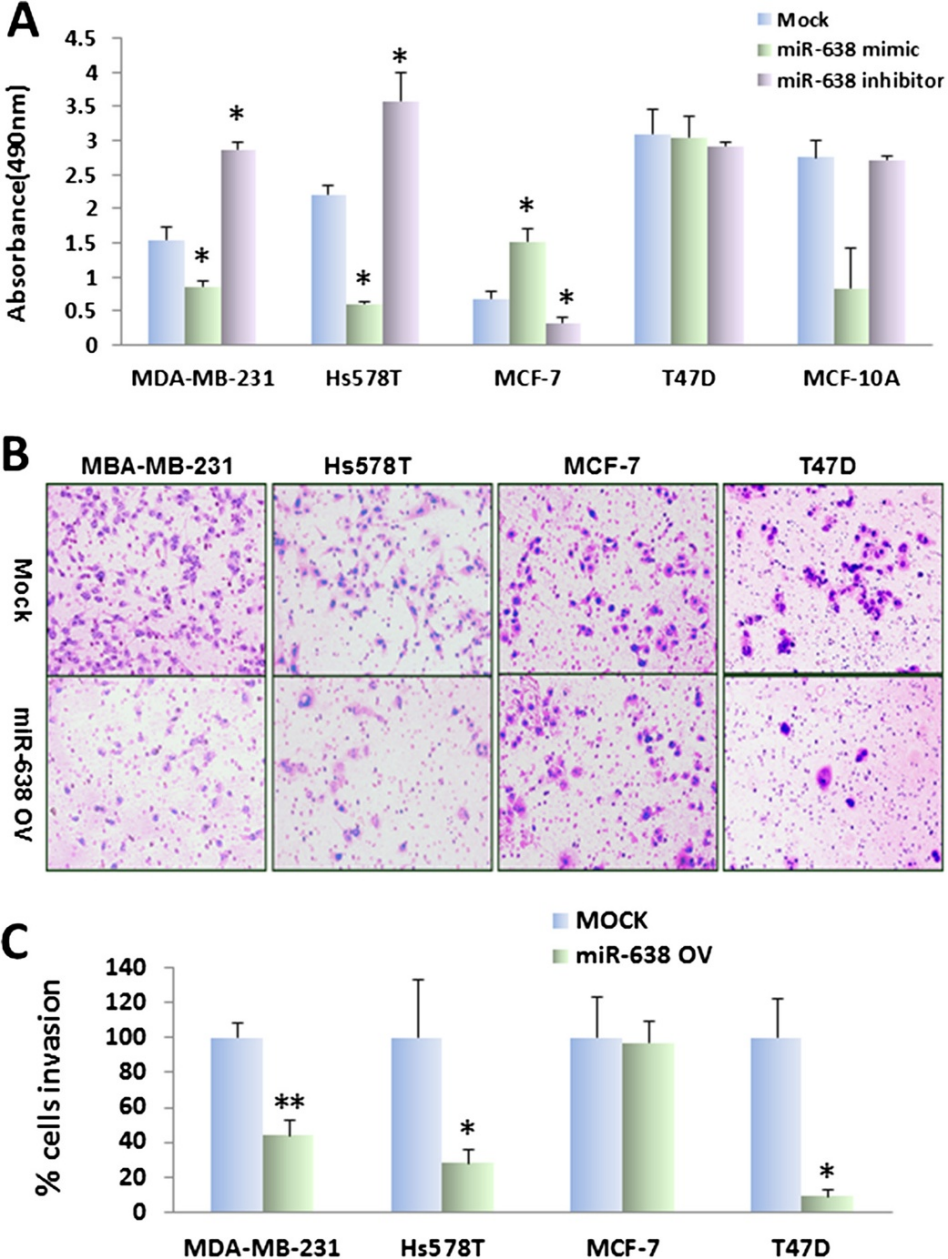
**miR-638 modulates proliferation in breast cancer cells and inhibits invasive ability in TNBC cells. (A)** Effects of miR-638 on cell proliferation were determined by MTT assay. The proliferation rate decreased after transfection of miR-638 mimic (gray bars) in TNBC cell lines MDA-MB-231 and Hs578T as well as in MCF-10A, and increased in MCF-7 cells, and had no effect in T47D cells, compared to the mock control (white bars). Transfection of miR-638 inhibitor (dark bars) promoted proliferation in TNBC cell lines, MDA-MB-231 and Hs578T, but inhibited proliferation in MCF-7 cells compared to the mock. There were no obvious changes in T47D cells in either mimic or inhibitor transfection, while MCF10A exhibited similar pattern as TNBC cells. Values represent the mean ± SE for three independent experiments. (^*^P <0.05). **(B)** Transwell assays with matrigel were performed for the invasion activity of breast cancer cells transfected with either miR-638 mimic or the mock control. Overexpression of miR**-**638 reduces cell invasion in TNBC cell lines, MDA-MB-231 and Hs578T, but not in ER-positive cells, MCF-7 and T47D. **(C)** Invasion ability of the cells was displayed as a percentage of the absolute cell numbers. Results are displayed as mean data ± SE. (^*^P <0.05 and ^**^P <0.001). Five fields of unit area on each membrane or whole membrane were counted for cell numbers, and the experiments were repeated three times in triplicate. ER, estrogen receptor; TNBC, triple-negative breast cancer.

Since the expression of miR-638 was significantly lower in both breast cancer cell lines and tissues compared to normal cells and tissues, we next focused on the functional effects of miR-638 on breast cancer cells. To address this, miR-638 mimic, miR-638 inhibitor or mock control was transfected into breast cancer cells. The cellular proliferation rate of breast cancer cell lines was determined by MTT assay. As expected, overexpression of miR-638 inhibited proliferation in TNBC cell lines, MDA-MB-231 and Hs578T, while increased cell growth in ER-positive MCF-7 cells, and had almost no effect in T47D cells compared to the mock-transfected control cells (Figure 3A). Conversely, transfection of miR-638 inhibitor resulted in an increased cell growth in MDA-MB-231 and Hs578T cells and a decreased cell growth in MCF-7 and no significant change in T47D cells. Surprisingly, decreased cell proliferation was also observed in miR-638-transfected MCF-10A cells, which are defined as `normal’ breast epithelial cells. However, there was no significant difference when miR-638 inhibitor was transfected into MCF-10A cells. These results demonstrate that miR-638 can inhibit cell proliferation in TNBC but not in ER-positive breast cancer cells, suggesting that overexpression of miR-638 may be a promising therapeutic option for TNBC. The downregulation of miR-638 could be a prognostic indicator for the aggressiveness.

### Overexpression of miR-638 affected invasion ability in breast cancer cell lines

To determine if overexpression of miR-638 affects the invasive ability of breast cancer cell lines, cell invasion assay was performed using the BD matrigel. We found that miR-638-overexpressing TNBC cell lines exhibited significant inhibition of invasion ability (60% in MDA-MB-231 and 70% in Hs578T) compared to the control. For ER-positive cell lines, miR-638-overexpressing MCF-7 cell line showed no invasion changes (*P* = 0.90) while T47D decreased its invasion activity, compared to the control (*P* = 0.47) (Figure 3B and C). This data suggests that miR-638 plays an important role in cell invasion, specifically in TNBC.

### Overexpression of miR-638 sensitizes the TNBC cell lines to DNA-damaging agents

**Figure 4:**
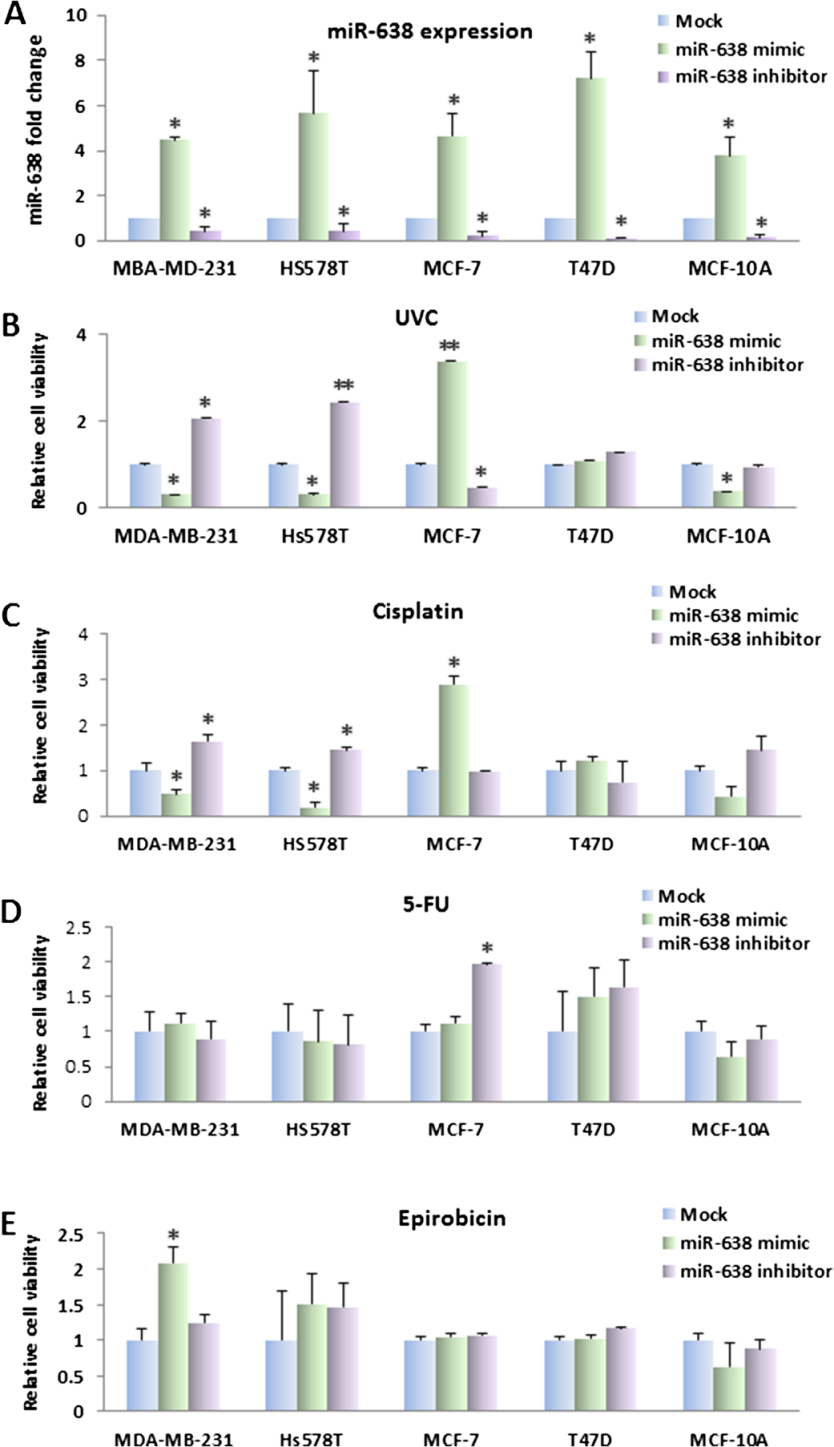
**Effect of miR-638 on sensitivity of breast cancer cell lines to anticancer drugs or UV treatment.** miR-638 expression in breast cancer cell lines transfected with miR-638 mimic or inhibitor compared to the mock control was examined by qRT-PCR **(A)**. Twenty-four h after transfection of miR-638 mimic, miR-638 inhibitor or mock, cells were treated by UVC, cisplatin, 5-FU and epirubicin, respectively for 48 h. Cell sensitivity was measured by MTT assay. miR-638 expression significantly increased sensitivity to UV **(B)** and cisplatin **(C)** in TNBC cell lines, MDA-MB-231 and Hs578T, but reduced sensitivity in MCF-7 cells compared with the mock. miR-638 expression did not change the sensitivity to 5-FU **(D)** and epirubicin **(E)** in breast cancer cell lines. Results are displayed as mean data ± SE. ^*^P <0.05, in comparison to the mock. 5-FU, 5-fluorouracil; TNBC, triple-negative breast cancer.

Overexpression of *BRCA1* in MCF7 cell line has been reported to result in an increased resistance to cisplatin [31]. Based on our evidence, we reasoned that overexpression of miR-638 could sensitize TNBC cells to DNA-damaging agents by means of their ability to reduce *BRCA1* expression and curb the activation of *BRCA1* in TNBC cells. To evaluate this hypothesis, we treated breast cancer cells transfected with miR-638 mimic or inhibitor (Figure 4A) with DNA-damaging agents (UVC, cisplatin and 5-FU) and non-DNA-damaging agents (epirubicin). UVC/chemosensitivity was determined by the MTT assay 48 hours after treatment. As shown in Figure 4B and C, miR-638 expression significantly increased sensitivity to UV and cisplatin in TNBC cell lines MDA-MB-231 and Hs578T, but reduced sensitivity in MCF-7 cells as compared with mock. Interestingly, a similar effect of cisplatin was observed in MCF-10A cells. The effect of miR-638 on sensitivity of UV and cisplatin was not observed in ER-positive cell line, T47D. Conversely, knockdown of miR-638 using miR-638 inhibitor increased cell viability in MDA-MB-231 and Hs578T cells after treatment with UV and cisplatin. It is notable that miR-638 introduction did not change the sensitivity to 5-FU and epirubicin in breast cancer cell lines (Figure 4D and E). These results suggested that the inhibition of miR-638 increased sensitivity to partial of DNA-damaging agents in TNBC but not in non-TNBC cells.

### miR-638 overexpression significantly reduced post-UV/drugs host cell reactivation activity in TNBC cells

**Figure 5:**
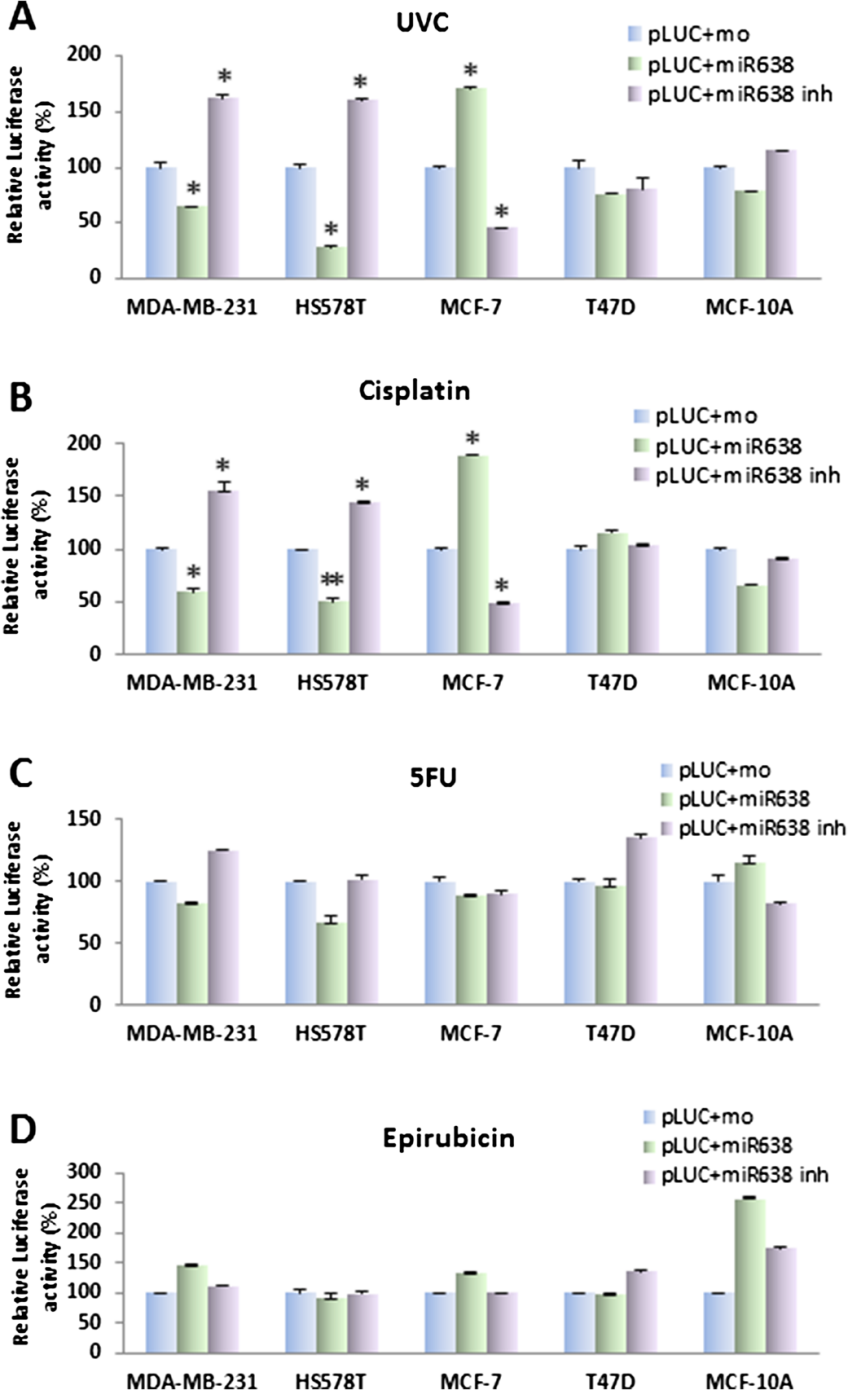
**Effects of miR-638 on DNA repair capability by host cell reactivation (HCR) assay.** Cells were transiently co-transfected by miR-638 mimic or inhibitor with pCMU-Luc vector pre-damaged by UVC, or anticancer drugs for the evaluation of DNA repair capacity. In **(A)** and **(B)**, MDA-MB-231 and Hs578T exhibited significantly reduced luciferase activity, while MCF-7 cell lines showed increased luciferase activity in miR-638 mimic-transfected cells, but there were no changes in T47D and MCF-10A when co-transfected with either miR-638 mimic or inhibitor with pCMU-Luc vector pre-damaged by UVC or cisplatin. **(C)** Cells were co-transfected with miR-638 mimic or inhibitor with pCMU-Luc vector pre-treated with 5-FU. Reduced DNA repair capability was only observed in Hs578T cells but not in MDA-MB-231, MCF-7, T47D and MCF-10A cell lines. **(D)** Cells were co-transfected with miR-638 mimic or inhibitor and pCMU-Luc vector pre-treated with epirubicin. No significant DNA repair capability changes were observed in all cell lines. Results are displayed as mean data ± SE (^*^P <0.05, ^**^P <0.01). 5-FU, 5-fluorouracil.

miR-638 is involved in DNA repair pathway regulation during carcinogen exposure [26]. *BRCA1* appears to promote cell survival after DNA damage by participating in repair pathways [32]. We then asked whether the deregulation of miR-638 increases the sensitivity of the TNBC cell lines to cisplatin and UV via the DNA repair pathway. We measured luciferase activity by co-transfecting miR-638 mimic or inhibitor, along with pCMU-Luc vector [29], which was pre-treated by UVC, DNA-damaging and non-DNA-damaging agents respectively into the breast cancer cell lines. For UV and cisplatin exposure, we found that TNBC cell lines MDA-MB-231 and Hs578T exhibited significantly reduced luciferase activity, while MCF-7 cell lines showed opposite effect. There were no changes in luciferase activity for T47D cells (Figure 5A and B). Conversely, contrary effect on DNA repair capability was observed in MDA-MB-231, Hs578T and MCF-7 cells after co-transfection of miR-638 inhibitor and the pCMU-Luc vector pre-treated with UVC and cisplatin. Our data indicates that miR-638 impairs the DNA repair capability by regulating *BRCA1* expression in TNBC cell lines.

For 5-FU exposure, reduced DNA repair capability was only observed in Hs578T cells but not in MDA-MB-231, MCF-7 and T47D cells when co-transfected with miR-638 mimic and pCMU-Luc vector pre-treated with 5-FU. No significant DNA repair capability changes were observed in cell lines when co-transfected with miR-638 mimic, and pCMU-Luc vector pre-treated with epirubicin. On the other hand, DNA repair capability did not change when co-transfected miR-638 inhibitor and pCMU-Luc pre-treated with either 5-FU or epirubicin. Our results demonstrate that the miR-638 exerts a distinct effect on DNA repair toward anticancer drugs in TNBC and ER-positive breast cancer cells.

## Discussion

The objective of this study was to decipher the role of miR-638 in breast cancer tumorigenesis and treatment. We revealed a dynamic miR-638 expression during the process of breast cancer progression. In addition, we demonstrated that different expression patterns of miR-638 are correlated with its functions in different types of breast cancer cells. Moreover, we found that the role of miR-638 on tumorigenesis, radiation and drug sensitivity was mediated by targeting *BRCA1* through DNA repair pathway. Our results suggest that aberrant expression of miR-638 contributes to human breast cancer progression, invasion and sensitivity of radiation and chemotherapy, particularly in TNBC.

### miR-638 expression changes during development of breast cancer

miRNA may exhibit different expression levels between normal and cancer cells [33]. For example, miR-142-3p and miR-9 were upregulated in squamous cell carcinoma in comparison to normal bronchial tissues. The alterations of miRNA expression were probably correlated with the pathways maintaining the malignant phenotypes or clinical outcomes [26]. miR-638 has been characterized to be downregulated in majority of tumors, but upregulated in hepatocellular liver cancer. Our previous data indicated that miR-638 was downregulated in ADH and IDC during the breast cancer developing stages compared to normal breast tissues [19]. In this study, we analyzed miR-638 expression in a new cohort of 30 breast cancer FFPE samples. We showed that downregulation of miR-638 presented in the majority of the IDCs compared to their adjacent normal tissues. These results support the notion that dysfunction of miR-638 is essential in maintaining the malignant phenotype of cancer. A larger cohort study will help understand the exact role of miR-638 in breast cancer development and progression. In addition, we assessed the expression of miR-638 in breast cancer cell lines. The expression of miR-638 was low in breast cancer cell lines compared with the MCF-10A cells (Figure 1C). Based on these data, we hypothesize that deregulation of miR-638 is involved in the process of breast cancer progression.

### miR-638 is a double-faced gene expression regulator

It is currently believed that miRNAs elicit their effect by silencing the expression of target genes [34]. However, miRNAs may also function to positively regulate gene expression [35],[36]. miR-638 has been reported to inhibit *BRCA1* expression in different cancer cell lines by targeting *BRCA1* in CDS, but not in 3’ UTR [30]. In our study, overexpression of miR-638 suppressed *BRCA1* expression in TNBC cells, MDA-MB-231 and Hs578T, but not in ER-positive cell lines. These data support the notion that miR-638 exerts diverse effects depending upon breast cancer types. Since TNBC lacks expression of *ER*, *PR* and *HER2*, we believe that hormones might be involved in miR-638-mediated *BRCA1* regulation, which requires further studies. Nicoloso *et al*. observed an opposite *BRCA1* regulation of miR-638 in MCF-7 cells [30]. A possible explanation might be that miR-638 exhibits distinct *BRCA1* regulation in cell populations with different phases of cell cycle. Our data suggests that miR-638 elicits differential efficacy by silencing or inducing the expression of *BRCA1* in different breast cancer cell lines. These findings demonstrate that miR-638 exhibits a dual function in response to environmental stimuli depending upon the state of cell malignancy. It might be a useful biomarker for surveillance of chemical exposure in breast cancer treatment.

### miR-638 plays an important role in cell proliferation and invasion

Previous studies have demonstrated that miR-638 regulates cell growth and smooth muscle cell proliferation and migration [37], and negatively regulates *BRCA1* expression. In esophageal squamous cell carcinoma, miR-638 promotes cell proliferation *in vitro*[23]. We analyzed the functional consequences after overexpressing miR-638 in breast cancer cell lines. Our data indicated that miR-638 has different functions on cell behaviors in different types of breast cancer cells. miR-638 inhibited cell proliferation in TNBC cells, while promoted cell proliferation in ER-positive cells. Interestingly, miR-638 exerts similar effect on inhibiting proliferation in MCF-10A, which is defined as `normal’ breast epithelial cells. Although MCF-10A is non-tumorigenic, it is a triple-negative human breast cell line [38]. Our data supports that miR-638 inhibits cell proliferation in TNBC.

Importantly, miR-638 also alters cell invasion ability. The invasion was decreased in TNBC cell lines after miR-638 overexpression, but increased in MCF-7 cells. We hypothesize that miR-638 functions as a tumor-suppressor or oncomir in different types of breast cancer cells or stages during breast cancer progression. However, miR-638 also suppresses the invasion in T47D cells although no significant downregulation of *BRCA1* was observed. The possibility might be that miR-638 regulates invasion in T47D via other pathways instead of *BRCA1-*related DNA repair pathway.

### miR-638 is associated with radiation and chemotherapy sensitivity of breast cancer cell lines via DNA repair pathway

miRNAs has been implicated in response to DNA damage and repair. Some miRNAs are involved in DNA damage response and/or DNA repair, which would affect cellular sensitivity to DNA-damaging agents [39]. In order to explore whether miR-638 expression could be used as a biomarker for predicting tumor response to chemotherapy and radiotherapy for breast cancer, we found that overexpression of miR-638 impaired DNA repair in breast cancer cell lines, which suggests that miR-638 might correspond to the cellular stress upon radiation and DNA-damaging agents.

## Conclusions

We previously found that miR-638 was one of the most deregulated miRNAs in breast cancer progression. In present work, we demonstrated that miR-638 directly regulates *BRCA1* expression in breast cancer, implying a critical role of miR-638 in the course of breast carcinogenesis and biological behaviors. miR-638 exerts distinct effects on cell proliferation and invasion in different types of breast cancer. In addition, miR-638 enhanced radiation and chemotherapy sensitivity in TNBC cells by regulating *BRCA1* expression via DNA repair pathways. Taken together, miR-638 may serve as a potential prognostic biomarker and therapeutic target for breast cancer.

## Authors’ contributions

XT contributed to the conception and design of the project, development of methodology, acquisition of data, analysis and interpretation of data, bioinformatics and statistical analyses, and writing of the manuscript. JP performed qRT-PCR and matrigel invasion assays, and helped write the manuscript. YF performed RNA isolation from FFPE samples and data analysis. SA performed cell culture, plasmids preparation and data analysis. KR, ST, CBT and YM identified pathological cases with clinical information and performed microdissection. RFB participated in the design of the project and the manuscript preparation. SWF designed and interpreted data, and wrote and revised the manuscript. All authors read and approved the final version of this manuscript.

## Authors’ original submitted files for images

Below are the links to the authors’ original submitted files for images. Authors’ original file for figure 1 Authors’ original file for figure 2 Authors’ original file for figure 3 Authors’ original file for figure 4 Authors’ original file for figure 5

## Abbreviations

5-FU: 5-fluorouracil
ADH: atypical ductal hyperplasia
*BRCA1*: breast cancer susceptibility gene 1
BSA: bovine serum albumin
CDS: coding sequence
DCIS: ductal carcinoma *in situ*
DMEM: Dulbecco’s modified Eagle’s medium
ER: estrogen receptor
FBS: fetal bovine serum
IDC: invasive ductal carcinoma
IgG: immunoglobulin G
FFPE: formalin-fixed, paraffin-embedded
HCR: host cell reactivation
MEGM: mammary epithelial cell growth medium
miRNAs: microRNAs
PBS: phosphate-buffered saline
TBS: Tris-buffered saline
TNBC: triple-negative breast cancer
UTR: untranslated region
