# Supplementary figures and images for: miR-638 mediated regulation of BRCA1affects DNA repair and sensitivity to UV and cisplatin in triple-negative breast cancer
Source: Breast Cancer Res. 2014 Sep 17;16:435. doi: 10.1186/s13058-014-0435-5 (PMC4303116; doi:10.1186/s13058-014-0435-5)

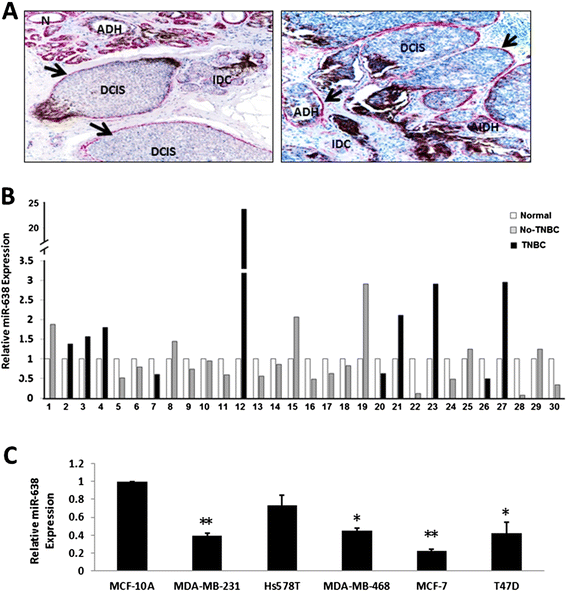

Supplement: Supplementary file 1 — Authors’ original file for figure 1 [file 13058_2014_435_MOESM1_ESM.gif]

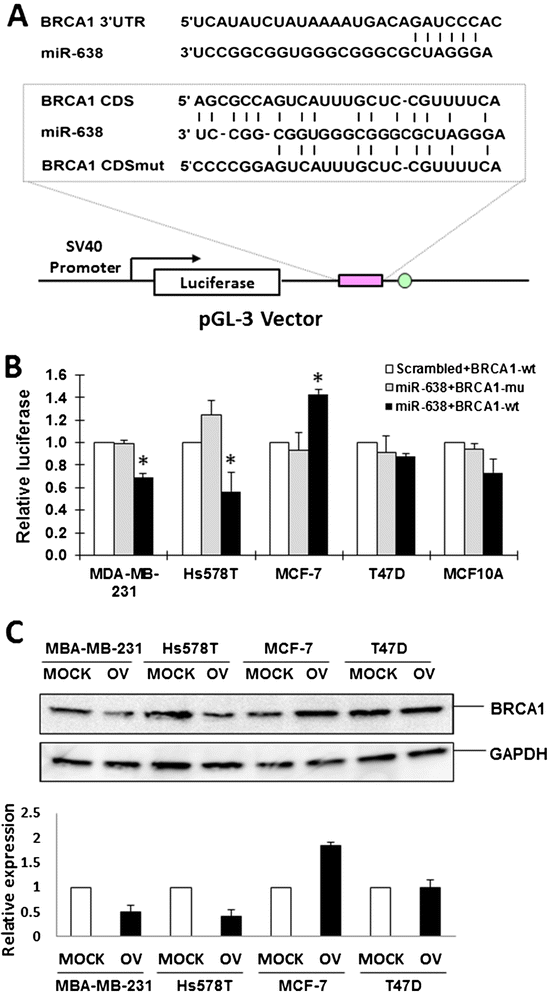

Supplement: Supplementary file 2 — Authors’ original file for figure 2 [file 13058_2014_435_MOESM2_ESM.gif]

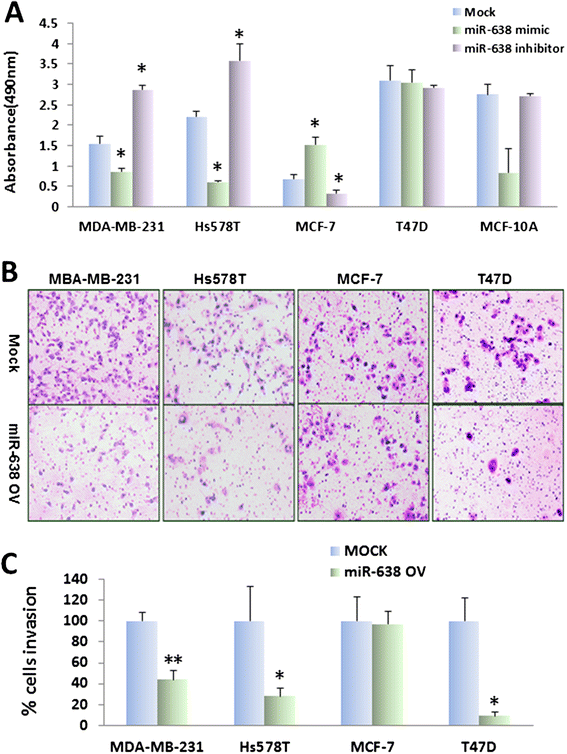

Supplement: Supplementary file 3 — Authors’ original file for figure 3 [file 13058_2014_435_MOESM3_ESM.gif]

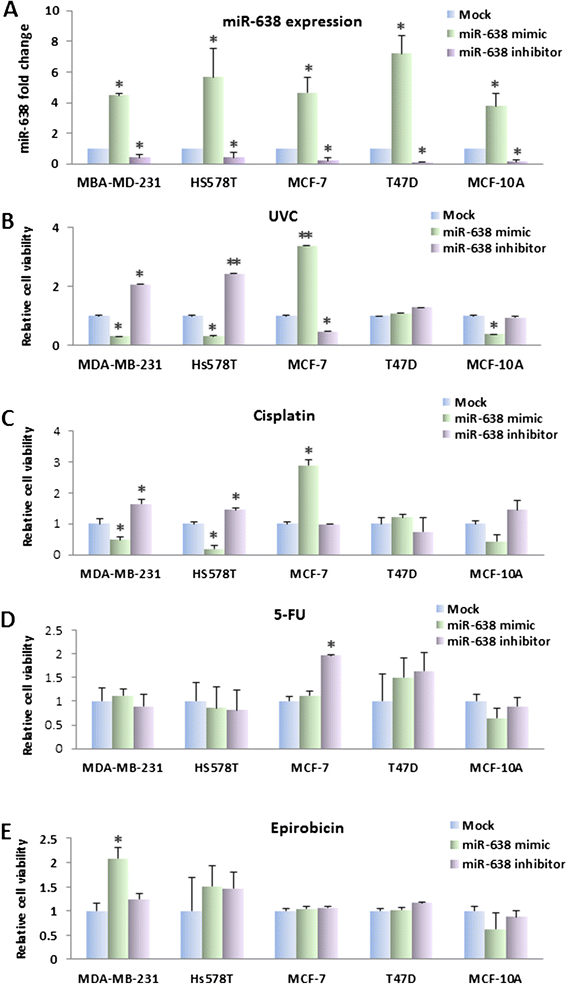

Supplement: Supplementary file 4 — Authors’ original file for figure 4 [file 13058_2014_435_MOESM4_ESM.gif]

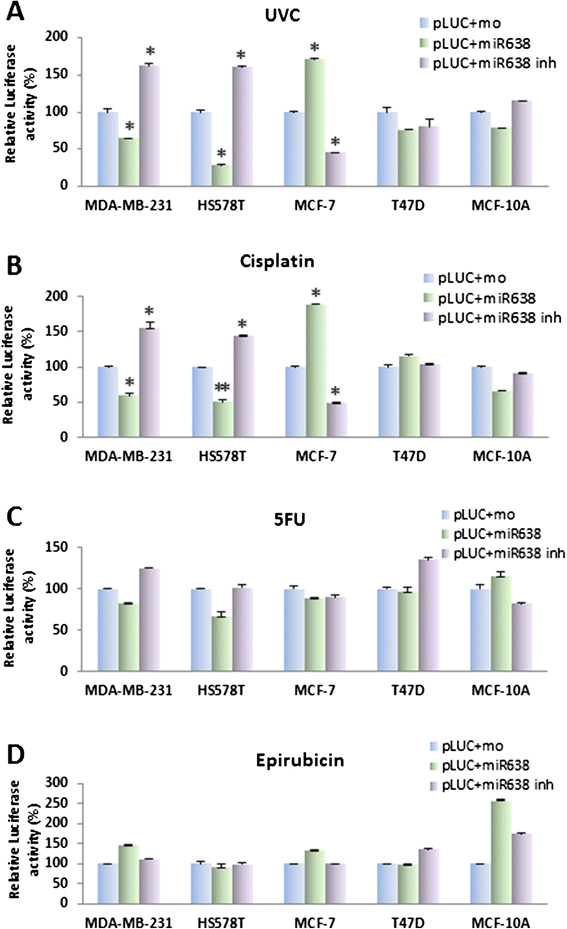

Supplement: Supplementary file 5 — Authors’ original file for figure 5 [file 13058_2014_435_MOESM5_ESM.gif]
